# A Novel Cause of Bowel Obstruction in a Patient with Long-Standing Crohn's Disease

**DOI:** 10.1155/2021/3278392

**Published:** 2021-10-18

**Authors:** Satya V. Vedula, T. Paul Nickerson, Douglas J. Grider

**Affiliations:** ^1^Virginia Tech Carilion School of Medicine, 2 Riverside Circle, Roanoke, VA, USA; ^2^Department of Surgery, Section of Colorectal Surgery, Virginia Tech Carilion School of Medicine, 2 Riverside Circle, Roanoke, VA, USA; ^3^Department of Basic Science Education, Virginia Tech Carilion School of Medicine, 2 Riverside Circle, Roanoke, VA, USA; ^4^Dominion Pathology Associates, 1 Riverside Circle, Roanoke, VA 24016, USA

## Abstract

Solitary fibrous tumors are rare tumors of mesenchymal origin. Although most often observed in the lung pleura, they have been reported in varied extrapleural sites. A 70-year-old male with complicated Crohn's disease presented with 3 days of nausea, emesis, constipation, and abdominal pain. Computed Tomography (CT) demonstrated mucosal thickening of the middescending colon, consistent with fibrosing stricture. Surgical excision revealed an unusual, 5 cm mass originating in the subserosa. Histopathology of the lesion was notable for a proliferation of cells with spindle and stellate-shaped nuclei and no appreciable mitotic figures, which extended into the muscularis and submucosa. Immunohistochemistry was STAT6 nuclear positive and cytoplasmic CD34 positive, diagnostic for solitary fibrous tumor (SFT). In this case, the SFT infiltrating into the muscularis propria and subserosa caused the stricture and bowel obstruction. This illustrates that while fibrosing strictures are usually the etiology of bowel obstruction in the setting of Crohn's disease, other rare possible causes should be considered.

## 1. Introduction

Solitary fibrous tumors (SFTs) are rare soft tissue tumors arising from spindle cells of mesenchymal origin [1]. A 70-year-old male with complicated Crohn's disease presented with symptoms of small bowel obstruction. Chronic cycles of inflammation and repair are the most common pathologic mechanism of fibrosing stricture causing bowel obstruction in the setting of Crohn's disease [2]. In this case, however, the eventual bowel obstruction was caused by an invasive SFT originating in the subserosa and extending to the muscularis propria and submucosa, which has not been reported in the literature to date. To our knowledge, this is also the first case of an SFT causing bowel obstruction directly, rather than by external compression of the bowel.

## 2. Case Presentation

A 70-year-old male with a long-standing history of complicated Crohn's disease presented to the Emergency Department (ED) with 3 days of nausea, emesis, abdominal pain, and constipation, consistent with prior bowel obstructions which had resolved with conservative management. Surgical history is significant for bilateral iliac artery angioplasty with transluminal stent placement 3 years prior to presentation. Current medications include loperamide, simethicone, and aspirin. Initial laboratory studies revealed mild anemia and slight hypokalemia but were otherwise unremarkable.

**Figure 1:**
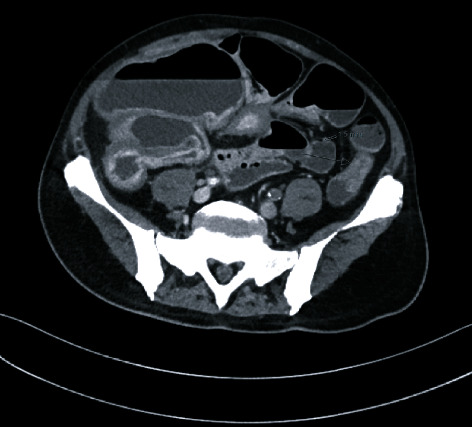
Abdominal CT scan taken at the L5 level, demonstrating focal mucosal thickening and dilation of the ascending and proximal descending colon.

Abdominal CT revealed focal mucosal thickening in the middescending colon, as well as dilation and mild mucosal thickening in proximal segments of the colon (Figure 1). A colonic stricture and consequent bowel obstruction were suspected, and the patient was admitted for exploratory laparotomy with lysis of adhesions and partial colectomy with diverting loop ileostomy. During surgery, a stricture was noted at the middescending colon at the observed location on CT and was extirpated.

**Figure 2:**
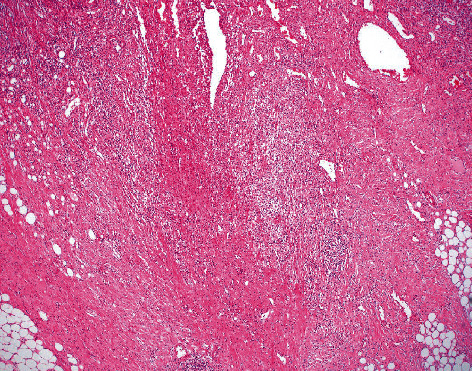
H&E demonstrating fissuring ulcer (tumor not in view) at 40 magnification (4x).

**Figure 3:**
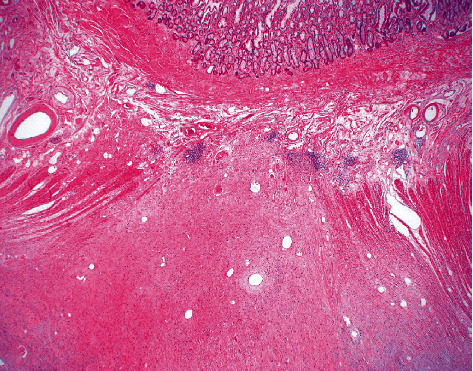
H&E demonstrating SFT with focal invasion of the submucosa at 20 magnification (2x).

**Figure 4:**
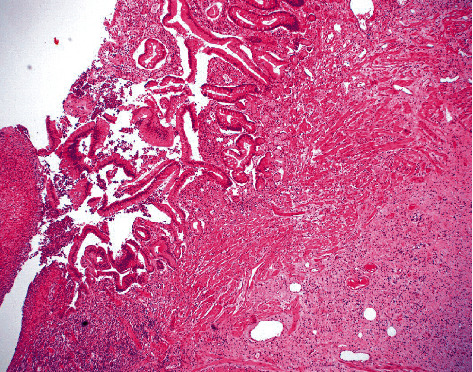
H&E from stricture site with tumor in view (bottom right) at 40 magnification (4x).

**Figure 5:**
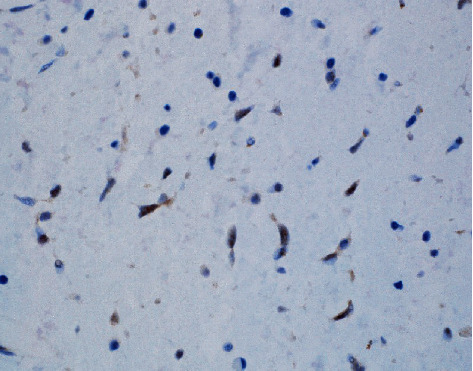
Nuclear STAT-6 positivity at 600 magnification (60x).

**Figure 6:**
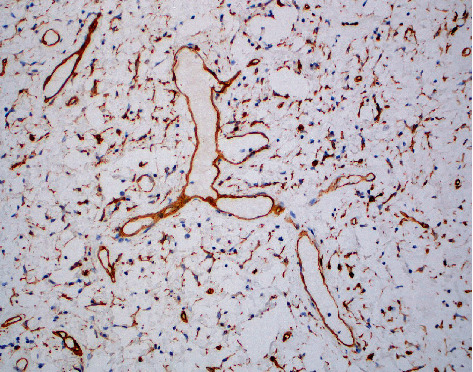
CD34 stain marking the cytoplasm of the stellate and spindle cells as well as a staghorn vessel at 200 magnification (20x).

Gross examination of the excised stricture revealed an unusual mass-like lesion. Histopathological examination of the surgical specimen revealed a fissuring ulcer typical for Crohn's disease (Figure 2) but also demonstrated a proliferation of cells with spindle to stellate shaped nuclei, intermixed mast cells, and no appreciable mitotic figures. This cell proliferation extended from the subserosa into the muscularis propria and focally into the submucosa at the area of fissuring, ultimately protruding into the bowel (Figures 3 and 4). The spindle/stellate cells were STAT-6 nuclear positive (Figure 5) and cytoplasmic CD34 positive (Figure 6). Blood vessels are also marked with CD34 (Figure 6). The lesion was negative for PDGFR-a mutations (exons 12 and 18). CD117 marked the background mast cells, but was negative in the tumor cells, excluding a gastrointestinal stromal tumor (GIST). Based on these histopathologic and immunohistochemical findings, the diagnosis of solitary fibrous tumor (SFT) was confirmed.

Unfortunately, the patient sustained acute lower limb ischemia due to incidental occlusion of his iliac artery stent, complicated by postoperative multisystem organ failure. He died 11 days after initial presentation.

## 3. Discussion

Solitary fibrous tumors (SFT) are rare soft tissue tumors that arise from spindle cells of mesenchymal origin and are comprised of small cells with spindle-to-stellate-shaped nuclei separated by thin bands of collagen and a mucinous stroma [1]. Although most commonly observed in the lung pleura, they have been reported in a variety of sites throughout the gastrointestinal tract and associated organs with varying effects on gastrointestinal function, including the esophagus, liver, pancreas, salivary glands, and mesentery, as well as in the spermatic cord, adrenal gland, prostate, and urethra [3–11].

On gross examination, SFTs are relatively nondescript, and must be differentiated from other tumors by histopathology and immunohistochemical examination. Differential diagnosis of SFT in the setting of Crohn's disease includes Inflammatory Fibroid Polyp (IFP), Gastrointestinal Stromal Tumor (GIST), Schwann cell proliferation, adenocarcinoma, and mucosal dysplasia. In particular, IFP has been reported as an occasional complication of inflammatory processes in patients with long-standing Crohn's Disease [12], and focal background blood vessels did show “onion-skin” like fibrosis, a common histological finding in IFP. However, other blood vessels were branched and “staghorn” in appearance (Figure 6) [1]. In addition, the lesion was subserosal in origin and not submucosal, supporting a diagnosis of SFT as IFP are submucosal in origin with no or minimal extension into the muscularis propria. Further, mutational analysis for the PDGFR-a gene was negative, excluding IFP, as these tumors typically demonstrate mutations to exon 12 or 18 in the PDGFR-a gene. GIST was excluded due to inconsistent histology and by a negative CD117 (*c-KIT)* stain. S100 stain was negative, excluding Schwann cell proliferations, such as neurofibroma or Schwannoma. While Crohn's disease may be associated with mucosal dysplasia and adenocarcinoma, neither were present in the surgical pathology specimen examined. Lastly, the spindled/stellate cells showed nuclear STAT-6-positivity (Figure 5) and cytoplasmic CD34-positivity (Figure 6), confirming SFT. While CD34 and Bcl-2 have high sensitivity for the detection of SFT [13], the presence of the *NAB2-STAT6* fusion gene is the most specific marker [14]. Benign SFTs are negative for CD117, desmin, smooth muscle actin, S100 protein, and inhibin, although malignant SFTs have been shown to be positive for S100, cytokeratin, vimentin, and p53 mutations as well [8]. This particular SFT was considered to be low-risk and nonmalignant due to small size (approximately 2 cm) and benign histology, including no appreciable mitotic figures.

Reports of SFTs originating in the bowel itself are rare, although a few cases of SFTs originating in the intestinal mesentery have been reported [7, 15, 16]. Liu et al. describe a case in which an SFT surgically excised from the small bowel mesentery was found to be responsible for a chronic abdominal mass, but the patient did not experience obstructive symptoms [7]. Santos et al. describe a case in which an SFT originating from the muscularis propria of the cecum was credited as the cause of hematochezia in an elderly patient, but likewise caused no signs of bowel obstruction [17]. Ligato et al. report a case in which an asymptomatic, 10 mm polyp was detected on routine colonoscopy and excised by snare polypectomy; subsequent immunohistochemical examination identified the structure as an SFT [18]. Finally, Bratton et al. describe a case in which an extrinsic sigmoid colon mass arising from the serosa was believed to reflect an adnexal mass in a 21-year-old female patient, causing acute constipation; it was later determined to be an SFT via postexcision immunohistochemistry [19]. However, this mass did not protrude into the bowel itself. Similar to the final case, most SFTs are clinically silent until they reach a size sufficient to compress surrounding structures.

In patients with complicated Crohn's disease, fibrosing strictures are a common pathologic mechanism for bowel obstruction. Chronic and persistent cycles of transmural injury, inflammation, and repair are believed to contribute to increased expression of TGF-*β* [20], increased extracellular matrix deposition [21], and activation of fibroblasts and myofibroblasts [22], ultimately leading to fibrosis and stricture. Indeed, our patient had a history of prior bowel obstructions, likely due to this mechanism. In this case, however, a nonmalignant, myxoid-type SFT invading the lumen of the small bowel was the cause of fibrosis and stricture, rather than the primary inflammation associated with Crohn's disease.

To our knowledge, there are no other cases in the literature of a solitary fibrous tumor causing bowel obstruction directly, rather than by external compression. This case also highlights that even in the setting of Crohn's disease, processes other than carcinoma or fibrosing stricture formation may contribute to Gastrointestinal Stromal Tumor bowel obstruction.

## 4. Conclusion

Solitary fibrous tumors are rare neoplasms arising from the mesenchyme that occur in a wide array of sites. SFTs typically cause clinical symptoms by way of external compression of organs, but this case highlights a novel patient in whom an SFT directly caused stricture and bowel obstruction. Although chronic inflammatory processes are the most common culprits of stricture and bowel obstruction in Crohn's disease patients, a broad differential diagnosis, including neoplasms such as SFT, is critical in the evaluation of these patients. While we have not observed any similar cases in the literature, this might be related to the rarity of SFTs in this setting, and the fact that such a process would be easily overlooked when the expectation is to find fibrosis.

## Data Availability

All data is available for review with Dominion Pathology Associates, Roanoke, Virginia.
